# Nitric Oxide Resistance in *Leishmania* (*Viannia*) *braziliensis* Involves Regulation of Glucose Consumption, Glutathione Metabolism and Abundance of Pentose Phosphate Pathway Enzymes

**DOI:** 10.3390/antiox11020277

**Published:** 2022-01-29

**Authors:** Nathalia Pinho, Ana Cristina Bombaça, Jacek R. Wiśniewski, Geovane Dias-Lopes, Leonardo Saboia-Vahia, Elisa Cupolillo, José Batista de Jesus, Roque P. de Almeida, Gabriel Padrón, Rubem Menna-Barreto, Patricia Cuervo

**Affiliations:** 1 Laboratório de Pesquisa em Leishmanioses, Instituto Oswaldo Cruz, Fiocruz, Rio de Janeiro 21040-360, RJ, Brazil; nathps11@gmail.com (N.P.); leovahia@gmail.com (L.S.-V.); elisa.cupolillo@ioc.fiocruz.br (E.C.); gpadronpalomares@gmail.com (G.P.); 2 Laboratório de Biologia Celular, Instituto Oswaldo Cruz, Fiocruz, Rio de Janeiro 21040-360, RJ, Brazil; anabombaca@gmail.com; 3 Biochemical Proteomics Group, Department of Proteomics and Signal Transduction, Max-Planck-Institute of Biochemistry, 82152 Planegg, Germany; jwisniew@biochem.mpg.de; 4 Laboratório de Biologia Molecular e Doenças Endêmicas, Instituto Oswaldo Cruz, Fiocruz, Rio de Janeiro 21040-360, RJ, Brazil; geovane.dl@gmail.com; 5 Departamento de Medicina, Universidade Federal de São João Del Rei, São João del Rei 35501-296, MG, Brazil; jbj@ufsj.edu.br; 6 Department of Medicine, Hospital Universitário, EBSERH, Universidade Federal de Sergipe, Aracaju 49100-000, SE, Brazil; roquepachecoalmeida@gmail.com

**Keywords:** *Leishmania braziliensis*, nitric oxide resistance, American Tegumentary Leishmaniasis (ATL), nitrosative stress, reactive oxygen species, reactive nitrogen species, quantitative proteomics, FASP, mass spectrometry, glycolysis, glutathione metabolism

## Abstract

In American Tegumentary Leishmaniasis production of cytokines, reactive oxygen species and nitric oxide (NO) by host macrophages normally lead to parasite death. However, some *Leishmania braziliensis* strains exhibit natural NO resistance. NO-resistant strains cause more lesions and are frequently more resistant to antimonial treatment than NO-susceptible ones, suggesting that NO-resistant parasites are endowed with specific mechanisms of survival and persistence. To tests this, we analyzed the effect of pro- and antioxidant molecules on the infectivity in vitro of *L. braziliensis* strains exhibiting polar phenotypes of resistance or susceptibility to NO. In addition, we conducted a comprehensive quantitative mass spectrometry-based proteomics analysis of those parasites. NO-resistant parasites were more infective to peritoneal macrophages, even in the presence of high levels of reactive species. Principal component analysis of protein concentration values clearly differentiated NO-resistant from NO-susceptible parasites, suggesting that there are natural intrinsic differences at molecular level among those strains. Upon NO exposure, NO-resistant parasites rapidly modulated their proteome, increasing their total protein content and glutathione (GSH) metabolism. Furthermore, NO-resistant parasites showed increased glucose analogue uptake, and increased abundance of phosphotransferase and G6PDH after nitrosative challenge, which can contribute to NADPH pool maintenance and fuel the reducing conditions for the recovery of GSH upon NO exposure. Thus, increased glucose consumption and GSH-mediated redox capability may explain the natural resistance of *L. braziliensis* against NO.

## 1. Introduction

*Leishmania* (*Viannia*) *braziliensis*, a New World Leishmania species, is an important etiological agent of American Tegumentary Leishmaniasis (ATL) in the Americas [1]. Infection with this species may have clinical outcomes, ranging from self-healing localized cutaneous lesions (LCL) to severe disseminated cutaneous and mucocutaneous forms that may result in facial mutilation by destruction of the palate and nose cartilage. Furthermore, disseminated cutaneous leishmaniasis (DL) and mucocutaneous leishmaniasis (MCL) caused by *L. braziliensis* are frequently refractory to treatment and result from the metastatic dissemination of the parasite [2]. Clinical manifestations result from both the host immune responses and the infecting parasite [3,4,5]. Indeed, intrinsic characteristics of parasites enable the subversion and/or immunomodulation of host immune responses, resulting in the inactivation of cell pathways crucial for parasite elimination [6,7,8,9].

Successful establishment of infection and further parasite persistence depend on a complex interaction between the *Leishmania*’s immune subversion arsenal and the microbicidal mechanisms of mononuclear phagocytes [10]. Such mechanisms include lysosomal enzymes, reactive oxygen species (ROS), and reactive nitrogen species (RNS). Oxidative burst and release of nitric oxide (NO) are the most effective mechanisms against *Leishmania* spp. [11,12]. ROS and RNS can be released when the macrophages are stimulated by TNF-α and IFN-γ, and their production is induced by the activation of routes that involve NADPH oxidases and inducible nitric oxide synthase (iNOS). When it happens, parasites are exposed to superoxide anion (O_2_^•−^), hydrogen peroxide (H_2_O_2_), peroxynitrite (ONOO^−^) and NO [13], which usually results in parasites elimination [11,14,15]. However, some *Leishmania* parasites present natural resistance to NO, helping them to escape the macrophage microbicidal responses and promoting their survival and persistence [16,17,18,19]. Indeed, *L. braziliensis* strains isolated from patients with different clinical forms of ATL exhibit different levels of natural resistance to NO. In vitro assays showed that amastigotes from NO-resistant strains survived and multiplied more in human macrophages. In addition, patients infected with NO-resistant parasites presented significantly more severe cutaneous lesions than those infected with NO-susceptible parasites [17].

In vitro infections of human macrophages with *L. braziliensis* NO-resistant strains showed a higher percentage of infected cells and reduced levels of TNF-α production than infections with NO-sensitive parasites. Furthermore, these NO-resistant strains were also correlated with higher refractoriness to pentavalent antimony, the first-line treatment for ATL, suggesting that NO resistance may be related to antimony resistance [20]. In addition, BALB/c mice infected with NO-resistant strains produce more IL-4, which stimulates increased expression of arginase-1, favoring parasites survival and severe forms of disease [21]. Additionally, *L. infantum* field isolates from relapse cases of visceral leishmaniasis are more resistant to antimonial and NO, as well as are more infective to macrophages in vitro, than parasites isolated from responsive patients [22]. However, the mechanisms by which *L. braziliensis* strains can resist/evade the nitrosative stress imposed by host cells have not been clearly defined. Potential mechanisms might include increased abundance of glucose-6-phosphate dehydrogenase (G6PDH), as shown in the study by 2DE-MS of the proteome of *L. infantum* strains resistant or susceptible to NO [23]. Nevertheless, an in-depth study of NO resistance in *Leishmania* is missing. 

Based on the previous evidence, we hypothesize that the proteome of *L. braziliensis* NO-resistant strains is tailored to deal with nitrosative stress and, that upon NO challenge, it can be rapidly regulated to minimize the damages caused by nitric oxide. Such adaptations grant the survival and persistence of parasites, leading to chronic infections that are refractory to treatment. To test this, we performed an unbiased and comprehensive quantitative proteomic analysis of NO-resistant and NO-susceptible *L. braziliensis* strains. By using previously reported procedures for parasite sample preparation and absolute label-free protein quantification [24,25], we were able to compare those parasites before and after stimulus with an NO donor, identifying ~6300 proteins and estimating absolute concentrations of ~6000 of these proteins. We also evaluated the effect of nitrosative and oxidative stresses, as well as the effect of antioxidant molecules, on the infection index of each strain. Together, our data provide new evidence on the potential mechanisms underlying the NO resistance in *L. braziliensis*, including increased antioxidant capability involving the glutathione (GSH) system and rapid regulation of glycolysis and pentose phosphate pathway (PPP). Data are available via ProteomeXchange with identifier PXD029462.

## 2. Materials and Methods

### 2.1. Ethics Statement

All the protocols were carried out in accordance with the recommendations of the Guide for the Care and Use of Laboratory Animals, according to resolution 196/96 of the National Council for Animal Experimentation—COBEA (https://sbcal.org.br/ (accessed on 2 March 2020)). All the procedures used in this study were approved by the Animal Use Ethics Committee of IOC/Fiocruz (L-005/2017). According to the Brazilian Law of Biodiversity, this study was registered at SisGen (AA2236F).

### 2.2. Parasite Culture and Growth Curve

The *L. braziliensis* strains IOC/L2853 (MHOM/BR/2004/LTCP 393) and IOC/L2856 (MHOM/BR/2003/LTCP 15171) used in this study were provided by the Collection of *Leishmania* of the Instituto Oswaldo Cruz (CLIOC, http://clioc.fiocruz.br/ (accessed on 2 March 2020)). The IOC/L2853 strain is resistant to NO and was isolated from a MCL patient, who was refractory to treatment. On the other hand, the IOC/L2856 strain is susceptible to NO and was isolated from LCL patient responsive to the treatment [17,20]. Both strains were isolated from the same geographical region and belong to the same zymodeme. Through the text, tables, and figures, those strains will be mentioned as 2853 and 2856.

Promastigotes were cultivated at 25 °C in Schneider’s medium (Vitrocell, Campinas, Brazil) supplemented with 20% fetal bovine serum (FBS; Vitrocell, heat-inactivated at 56 °C for 50 min) and 2% urine. To analyze the growth curve of these strains, promastigotes (1 × 10^5^ parasites) were incubated in the culture medium described above, and parasite density was determined every 24 h during 16 days by counting in hemocytometer under light microscopy. Parasites of three-days-old culture (log phase promastigotes) were used for all experiments. The in vitro passages of parasites were controlled, and parasites’ infectious capacity was maintained through inoculation in golden hamsters.

### 2.3. Inhibitory Activity of NO Donor on Promastigotes of L. braziliensis

To evaluate the cytotoxicity of NaNO_2_ (NO donor) on NO-resistant (2853) or NO-susceptible (2856) *L. braziliensis* strains, promastigotes were resuspended in Hanks balanced solution (HBBs, pH 5.0; Sigma-Aldrich, St. Louis, MO, USA), and 100 μL (2 × 10^7^ protozoa/mL) was added to the same volume of freshly NaNO_2_ previously prepared at twice the desired final concentrations (32 to 0.06 mM). Microplates were incubated at 25 °C for 4 h. Together with the NO donor, 20 µL of PrestoBlue (Invitrogen, Carlsbad, CA, USA) was added to the final concentration of 10%. The measurements were performed at 560 and 590 nm, as recommended by the manufacturer at SpectraMax M3 fluorimeter (Molecular Devices, San Jose, CA, USA), and the result was expressed as IC_50_/4 h, which corresponds to concentration that led to 50% lysis of the parasites within 4 h. 

### 2.4. Effect of Pro-Oxidant and Antioxidant Molecules on Intracellular Amastigotes

To analyze the effect of oxidative burst on the infection, macrophages were collected from the peritoneal cavity of uninfected male BALB/c mice (5–6 weeks) after the injection of 10 mL of RPMI medium (LGC Biotecnologia, Cotia, Brazil). Peritoneal macrophages were resuspended in RPMI supplemented with 10% FBS and plated in 24-well plates (3 × 10^5^ cells/well) for 24 h at 37 °C and 5% CO_2_. After that, culture medium was replaced, and cells were infected or not with promastigotes (10:1 parasites/host cell) of each strain. After 4 h of incubation, cultures were washed to remove non-internalized parasites and maintained under the same conditions until complete 24 h of infection. Infected macrophages were treated or not with 150 µM hydrogen peroxide (H_2_O_2_), 2 mM NaNO_2_, 30 U/mL superoxide dismutase from bovine erythrocytes (SOD; Sigma-Aldrich, St. Louis, MO, USA), 40 U/mL catalase from bovine liver (Sigma-Aldrich, St. Louis, MO, USA), 1 µM mitoTEMPO (Santa Cruz Biotechnology, Dallas, TX, USA), or 2 mM Nω-Nitro-L-arginine methyl ester hydrochloride (L-NAME; Sigma-Aldrich, St. Louis, MO, USA), for 48 h. The culture was then stained with fast panoptic (Laborclin, Pinhais, Brazil), and percentage of infected cells, number of protozoa per cells, and infection index (percentage of infected host cells multiplied by the number of parasites per cell) were calculated. 

As host toxicity control, non-infected macrophages (5 × 10^4^ cells/well) were also treated with the compounds for 48 h at 37 °C and 5% CO_2_. After the treatment, 10 µL PrestoBlue was added to the final concentration of 10% and cells were incubated for 2 h at 37 °C and 5% CO_2_. The measurements were performed at 560 and 590 nm in a SpectraMax M3 fluorimeter.

### 2.5. ROS and RNS Release by Infected Macrophages

To evaluate the levels of reactive species released from infected cells, peritoneal macrophages were obtained as described above and incubated with LPS (100 ng/mL) plus IFN-γ (10 ng/mL) for 30 min. Cells were then infected for 72 h, and culture supernatants were collected for RNS detection by Griess reagent (Sigma-Aldrich, St. Louis, MO, USA) according to manufacturer’s instructions. Briefly, 100 µL of culture supernatant was added to the same volume of colorimetric reagent and incubated at room temperature for 30 min, and absorbance was read at 540 nm. Values were compared against an NaNO_2_ standard curve, ranging between 50 and 3 µM. In parallel, macrophages were resuspended in 100 µL respiration buffer consisting of 65 mM KCl, 10 mM Tris-HCl (pH 7.2), 1 mM MgCl_2_, and 2.5 mM potassium phosphate monobasic [26]. After that, 100 µM Amplex Red reagent (Invitrogen, Carlsbad, CA, USA) and 50 U/mL horseradish peroxidase (HRP; Sigma-Aldrich, St. Louis, MO, USA) were added and the cells were incubated for 30 min at 37 °C. The supernatants were then collected and analyzed to ROS presence by a SpectraMax M3 fluorimeter. 

### 2.6. Protein Extraction and Sample Preparation

For proteomic analysis, promastigotes were treated or not with ^1^/_5_ IC_50_/4 h NaNO_2_ (for each strain) in HBSS for 4 h at 25 °C. Then, parasites were washed three times with PBS and resuspended in lysis buffer for proteomics sample preparation. Because we are interested in the early effects of NO on proteome remodeling, we choose a sublethal dose to challenge the parasites. Additionally, with this way we avoid results related to parasites’ death. These assays were performed in quadruplicate (four independent biological replicates), and samples were prepared accordingly to previous reports [25]. Briefly, parasites were lysed in a buffer containing 0.05 M Tris-HCl (pH 7.6), 0.05 M DTT and 2% SDS (*w*/*v*) and boiled in a water bath for 5 min. After chilling to room temperature, the SDS lysates were clarified by centrifugation at 10,000×g for 5 min and processed in 30k filtration units (Millipore, Burlington, MA, USA) using the MED-FASP (Multi-Enzyme Digestion—Filter Aided Sample Preparation) protocol. Proteins were digested sequentially with endoproteinase LysC and trypsin in a 1/100 enzyme to protein ratio [27,28]. Peptides were collected, concentrated, and desalted on a C18 reversed phase column. 

### 2.7. LC-MS/MS Analysis

Analysis of the peptide mixtures was performed as described previously [25]. Briefly, the peptides were fractionated on a reversed phase column (50 cm × 75 μm inner diameter) packed with 1.8 μm diameter C18 particles (100 Å pore size; Dr. Maisch, Ammerbuch-Entringen, Germany) using a 105 min acetonitrile gradient in 0.1% formic acid at a flow rate of 250 nL/min. Peptide masses were analyzed using a Q-Exactive HF mass spectrometer (Thermo-Fisher Scientific, Palo Alto, CA, USA) operated in data-dependent mode with survey scans acquired at a resolution of 50,000 at *m*/*z* 400 (transient time 256 ms). Fifteen of the most abundant isotope patterns with charge ≥ +2 from the survey scan (300–1650 *m/z*) were selected with an isolation window of 1.6 *m/z* and fragmented by HCD with normalized collision energies of 25. The maximum ion injection times for the survey scan and the MS/MS scans were 20 and 60 ms, respectively. The ion target values for MS1 and MS2 scan modes were set to 3 × 10^6^ and 1 × 10^5^, respectively. The dynamic exclusion was 25 s and 10 ppm.

### 2.8. Data Analysis

The mass spectra were searched against a database containing *L. braziliensis* sequences available at UniProtKB/Swiss-Prot, using the Andromeda search engine included in the MaxQuant Software (Ver. 1.2.6.20). Reversed proteins were used as decoys. The option “matching between runs” was used for searching, and parameters such as fragment ion mass tolerance of 0.5 Da and parent ion tolerance of 20 ppm were included. Cysteine carbamidomethylation was set as fixed modification, methionine oxidation as variable modification, and up to two missed cleavages were allowed. The maximum false peptide and protein discovery rate was set as 1%. Protein absolute abundances were calculated based on the spectral protein intensity (raw intensities) using the ‘Total Protein Approach’ (TPA), and absolute protein copy numbers per cell were estimated using the ‘Proteomic Ruler’ approach [29]. Calculations of total protein, protein concentration, and copy number were performed in Microsoft Excel. Perseus software (Ver. 1.6.5.0) [30] was used for statistical analysis. Minimal number of valid values was set to 4 per protein in at least one group, and missing values were imputed from a normal distribution. Significances were calculated using the t test for pairwise comparisons, with a threshold of false discovery rate (FDR) of 3%. The mass spectrometry proteomics data were deposited to the ProteomeXchange Consortium via the PRIDE [31] partner repository with the dataset identifier PXD029462.

### 2.9. Analysis of 2-NBDG Uptake

Promastigotes (5 × 10^6^ protozoa/mL) were treated with ^1^/_5_ IC_50_/4 h NaNO_2_ in HBSS at 25 °C for 4 h. After that, protozoa were washed with PBS and incubated with 300 µM 2-(N-(7-Nitrobenz-2-oxa-1,3-diazol-4-yl)Amino)-2-Deoxyglucose) (2-NBDG, Molecular Probes, Eugene, OR, USA), a fluorescent glucose analogue, for 30 min. The 2-NBDG specificity was monitored by the parasite incubation at 4 °C for the same amount of time, to decrease the compound uptake. 2-NBDG+ parasites were analyzed using a CytekDxP multi-color upgrade flow cytometer (Cytek, Fremont, CA, USA). A total of 10,000 events were acquired in the region previously established by protozoa morphology, and analyses were performed in Summit 6.1 software (Beckman Coulter, Brea, CA, USA).

### 2.10. Analysis of Oxygen Uptake

Promastigotes (5 × 10^6^ protozoa/mL) were treated with ^1^/_5_ IC_50_/4 h NaNO_2_ in HBSS at 25 °C for 4 h. After that, protozoa were washed with PBS and resuspended (2.5 × 10^7^ protozoa/mL) in a respiration buffer at 25 °C with continuous stirring in a high-resolution Oxygraph-2K (Oroboros Instruments, Innsbruck, Austria) [26]. Mitochondrial respiration was confirmed by the addition of 2 µM antimycin A (AA, Sigma-Aldrich, St. Louis, MO, USA) to obtain the residual O_2_ consumption (ROX). O_2_ concentration and flux data were acquired using DatLab software (Oroboros Instruments, Innsbruck, Austria).

### 2.11. Analysis of Enzymatic Activities

Promastigotes (5 × 10^6^ protozoa/mL) were treated with ^1^/_5_ IC_50_/4 h NaNO_2_ in HBSS at 25 °C for 4 h, washed with PBS, and kept dry at −20 °C until use to ensure that all biological replicas were extracted and analyzed at the same time and under the same experimental conditions. Protein homogenates were prepared by sonication, as previously described [32]. Briefly, pellet was resuspended in cold PBS containing protease inhibitor cocktail (Sigma-Aldrich, St. Louis, MO, USA) and disrupted on ice by sonication for 10 cycles of 7 s with intervals of 7 s, using a Markson GE50 Ultrasonic Processor. Amplitude was set to 70%, and parasite disruption was monitored by light microscopy. Soluble fraction was obtained, and protein concentration was measured using Pierce™ BCA protein assay kit (Thermo Fisher Scientific, Palo Alto, CA, USA). For glutathione peroxidase (GPx) assay, soluble protein extract (0.5 mg/mL) was incubated in 100 mM KH_2_PO_4_ (pH 7.8) buffer, supplemented with 1 mM reduced glutathione (Sigma-Aldrich, St. Louis, MO, USA), 5 U/mL glutathione reductase (Sigma-Aldrich, St. Louis, MO, USA), 200 µM NADPH (Sigma-Aldrich, St. Louis, MO, USA), and 300 µM H_2_O_2_. The rate of the NADP (ε = 6.22 M^−1^cm^−1^) reduction was determined at 340 nm. Lactate dehydrogenase activity was evaluated using a commercial kit (Doles, Goiânia, Brazil) following the manufacturer’s protocol, with some modifications. Briefly, 0.5 mg/mL of protein was added to 100 µL manufacturer’s substrate solution and 10 µL ferric alum for 2 min at 37 °C. After that, 10 µL NAD+ + phenazine methosulfate (PMS) was added, and absorbance measured for 45 min (every 1 min) at 510 nm. A standard curve was generated with lactate dehydrogenase (Sigma-Aldrich, St. Louis, MO, USA). All enzyme activities were measured at 37 °C in a total reaction volume of 200 µL using a SpectraMax Plus384 spectrophotometer (Molecular Devices, Sunnyvale, CA, USA).

### 2.12. Statistical Analysis

Analyses were performed with GraphPad Prism version 8.0 for Windows (GraphPad Software, San Diego, CA, USA) or IBM SPSS Statistics 22.0 software (IBM Corporation, New York, NY, USA). Asterisks indicate significant differences with the threshold for significance set at *p* ≤ 0.05. Student’s t test or two-way ANOVA were used to analyze the statistical significance between the strains. The pairwise comparisons and the number of biological replicates are described in figure legend.

## 3. Results

### 3.1. The NO-Resistant 2853 L. braziliensis Strain Is More Infective to Macrophages In Vitro

**Figure 1:**
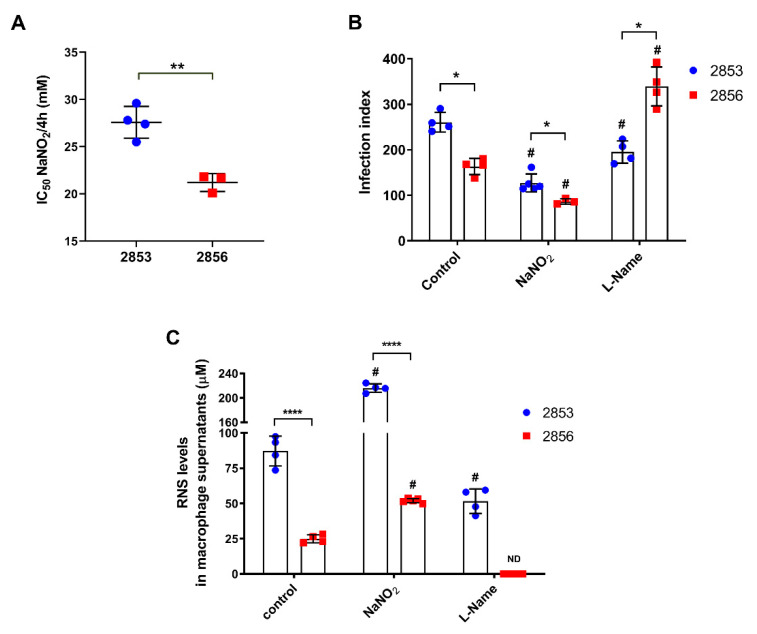
In vitro evaluation of *L. braziliensis* NO resistance and RNS levels under nitrosative stress. (**A**) Parasites naturally resistant (2853 strain) or susceptible to NO (2856 strain) were exposed to NaNO_2_ (ranging 0.06–32 mM) during 4 h. Values correspond to the NaNO_2_ concentration that reduces 50% parasite viability at 4 h of exposure. Dot plots represent mean ± SD of four independent experiments. Statistical differences by t test (** *p* = 0.002). (**B**) Peritoneal macrophages obtained from BALB/c mice were infected for 24 h with promastigotes of each strain. After that, cells were treated with 2 mM NaNO_2_ or 2 mM L-NAME for 48 h, completing 72 h of infection. Controls correspond to untreated cells infected for 72 h; infection index = percentage of infected host cells × number of parasites per 100 cells. (**C**) Macrophage supernatants were collected, and RNS levels were analyzed by Griess Reagent according to manufacturer’s instructions. Graphs represent mean ± SD of at least three independent experiments. Significance of differences between strains were determined by t test using the Holm–Sidak method for multiple comparisons (* *p* < 0.05; **** *p* < 0.0001). Significance of differences between treatments in comparison to control were determined by two-way ANOVA followed by Dunnett’s multiple comparisons test (# *p* ≤ 0.01); ND = no detected.

To verify the difference of resistance or susceptibility to NO of the two previously characterized *L. braziliensis* strains [17,20], the inhibitory concentration (IC_50_/4 h) of NaNO_2_ (NO donor) was evaluated in vitro using replicative promastigotes (logarithmic phase), collected at third day of growth (Figure S1). Confirming the previously reported phenotypes, 2853 strain was significantly more resistant to NO than 2856, exhibiting IC_50_/4 h of 27.6 ± 1.7 and 21.2 ± 0.9 mM, respectively (Figure 1A). The infection index of peritoneal macrophages, which corresponds to percentage of infected host cells × number of parasites per 100 cells, was significantly higher with the NO-resistant parasites (2853) than with the NO-susceptible (2856), even after the treatment of infected macrophages (24 h post-infection) with the 2 mM NaNO_2_. Additionally, the treatment with 2 mM L-NAME, a specific NOS inhibitor, led to a significant increase of the infection index observed in 2856-infected macrophages, in comparison to 2853-infected ones (Figure 1B). 

Furthermore, analysis of peritoneal macrophages supernatants showed that both *L. braziliensis* strains triggered NO-mediated response in infected cells; however, the RNS levels were significantly higher after the infection with NO-resistant parasites, reaching an increase of 3.5-fold in comparison to NO-susceptible strain. Treatment with NaNO_2_ also potentiated the nitrosative stress started by parasite infection, with such increase being more pronounced in 2853-infected cells. Interestingly, treatment with the NOS inhibitor decreased the production of RNS in both infections, abolishing its detection in the supernatant of 2856-infected cells but not in 2853-infected ones (Figure 1C).

### 3.2. NO-Susceptible Parasites Trigger a More Intense ROS-Dependent Response in Peritoneal Macrophages

**Figure 2:**
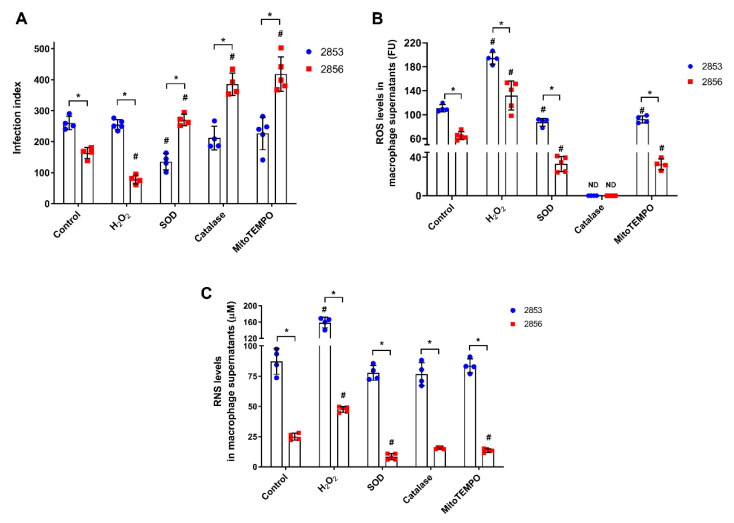
Effect of ROS and RNS levels in peritoneal macrophages infected in vitro with *L. braziliensis* strains resistant and susceptible to NO. Peritoneal macrophages obtained from BALB/c mice were infected with promastigotes for 24 h. After that, cells were treated with 150 µM H_2_O_2_, 30 U/mL SOD, 40 U/mL catalase, or 1 µM mitoTEMPO for 48 h, completing 72 h of infection. Controls correspond to untreated cells infected for 72 h. (**A**) Infection index, which corresponds to percentage of infected host cells × number of parasites per 100 cells. (**B**) Host cells were incubated with 100 µM Amplex Red and 50 U/mL HRP for 30 min in respiration buffer and analyzed for detection of ROS levels. (**C**) Macrophage supernatants were collected, and the production of RNS was analyzed by Griess Reagent, according to manufacturer’s instructions. Graphs represent mean ± SD of at least four independent experiments. Significance of differences between strains were determined by *t* test using the Holm-Sidak method for multiple comparisons (* *p* ≤ 0.01). Significance of differences between treatments in comparison to control was determined by two-way ANOVA followed by Dunnett’s multiple comparisons test (# *p* ≤ 0.05); ND = no detected.

Since nitrosative and oxidative stresses work together for host cell’s microbicidal mechanisms [13], we evaluate whether resistance to NO in *L. braziliensis* strains can influence the infective capacity after oxidative burst. To test this, infected peritoneal macrophages were treated with 150 µM H_2_O_2_ and several antioxidants for 48 h. Treatment with the pro-oxidant molecule significantly decreased the infection index observed in NO-susceptible group, amplifying the differences found between the infections caused by 2853 and 2856. On the other hand, the antioxidants presence increased the infection by NO-susceptible parasites, leading 2856 strain to reach infection index higher than those observed with the NO-resistant parasites (Figure 2A). Treatment with H_2_O_2_ also decreased by 52.2% the infection caused by the NO-susceptible parasites in comparison to control group, while antioxidants tested increased up to 2.5-fold the infection index of 2856-infected cells. In contrast, neither H_2_O_2_ nor most of the antioxidant molecules altered the infection index by NO-resistant strain; only the incubation with SOD decreased by 48% the 2853-infection index in relation to control (Figure 2A). 

Besides RNS, both *L. braziliensis* strains also trigger ROS-mediated responses in peritoneal macrophages; however, ROS levels were 1.7-fold higher in 2853-infected cells than in 2856-infected ones (Figure 2B). As expected, the treatment with H_2_O_2_ increased ROS levels in supernatants of peritoneal macrophages (up to 2-fold), and antioxidant molecules significantly decreased the oxidative burst in all analyzed conditions. Nevertheless, even in the presence of antioxidants, ROS levels were significantly higher (~3-fold) in macrophages infected with the NO-resistant strain than in those infected with the NO-susceptible. Interestingly, the treatment with catalase was able to abolish completely the H_2_O_2_ detection in both infection conditions (Figure 2B). 

To test the existence of a relation between the oxidative and nitrosative metabolism during *L. braziliensis* infection, supernatants of those peritoneal macrophages were also evaluated regarding to RNS levels. Remarkably, in all conditions, after treatment with H_2_O_2_ or antioxidants, macrophages infected with the NO-resistant strain produce among ~3.3-fold to ~9-fold higher levels of RNS than those infected with the NO-susceptible strain (Figure 2C). Treatment with H_2_O_2_ significantly increased the production of RNS (up to 1.9-fold) in cells infected by both strains, in comparison to infected control group, while antioxidants modulated RNS production only in 2856-infected macrophages, decreasing nitrosative levels. Interestingly, macrophages infected with the 2853 did not suffer such modulation, maintaining high levels of RNS even in the presence of antioxidant molecules (Figure 2C). 

### 3.3. Difference in Protein Abundance Is Observed between NO-Resistant and NO-Susceptible L. braziliensis Strains and it Is Significantly Modulated in Response to NO

As we observed that the NO-resistant strain was significantly more resistant to NO and more infectious for the host cells even after treatment with H_2_O_2_ and NaNO_2_, we decided to evaluate the protein abundance profile of this strain with and without NaNO_2_ challenge and compare it to the profile of the NO-susceptible strain. Mass spectrometry analysis of four biological replicates (independent biological assays) of each strain, challenged or not with ^1^/_5_ IC_50_/4 h NaNO_2_, allows for the identification of 6296 protein groups, encompassing ~80% of the *Leishmania* predicted proteome (~8000 protein-coding genes predicted, considering one protein per gene) (Table S1). More than 5700 protein groups were identified in each replicate, and 5010 protein groups were identified in all 16 samples (Table S2). 

**Figure 3:**
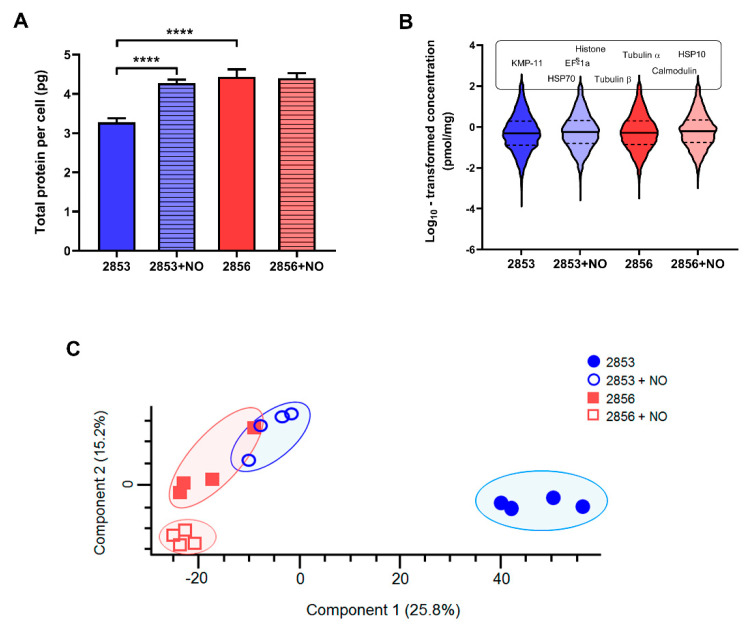
NO-resistance modulates protein abundance of *L. braziliensis* strains. Whole cell lysates from 2853, 2853+NO (2853 challenged with the ^1^/_5_ IC_50_/4 h NaNO_2_), 2856, and 2856+NO (2856 challenged with the ^1^/_5_ IC_50_/4 h NaNO_2_) strains were processed by MED-FASP and analyzed by LC-MS/MS. Protein abundances were calculated based on the raw spectral intensities. (**A**) Total protein content per cell of four biological replicates for each strain. Graphs represent mean ± SD of four independent experiments. Statistical differences by *t* test (**** *p* < 0.0001). (**B**) Violin plots depicting distribution of log10 transformed protein concentration values of proteins identified and quantified in each strain. Protein names above violin plots represent most abundant proteins common for all strains. (**C**) Principal component analysis of the absolute protein concentration values of all quantified proteins determined by the Total Protein Approach (TPA) method.

The total protein contents per cell were calculated as previously described [25] using the histone ruler method based on the DNA content reported for *L. braziliensis* reference (strain M2904). Similar to the estimates previously reported [25,33], the 2853 strain contains 3.3 ± 0.11 pg of protein per cell. Remarkably, the protein content of this strain increased significantly when parasites were challenged with the NO donor, reaching 4.3 ± 0.08 pg of protein per parasite (Figure 3A). Interestingly, the 2856 strain exhibited 4.4 ± 0.2 pg of total protein per cell, and this value did not change after challenge with NO (4.4 ± 0.1 pg) (Figure 3A).

Using the total protein approach (TPA) method, we calculated the absolute protein concentrations for each strain. In agreement with previous reports [25], we observed that protein concentration values span 6 orders of magnitude; 90% of the proteome extends over ~3 orders of abundance; and histones, alpha- and beta-tubulin, elongation factor 1-alpha, HSP70, and calmodulin are among the top 20 most abundant proteins (Figure 3B). Remarkably, principal component analysis (PCA) of the protein concentrations revealed a consistent separation between the NO-resistant and NO-susceptible strains, as well as between these strains after the NO challenge (2853+NO and 2856+NO) (Figure 3C), showing a clear clustering for each set of biological replicates. Statistical analysis by PCA also showed that the proteome of the NO-resistant strain is clearly modulated, in terms of protein concentrations, after the NaNO_2_ challenge.

Using Perseus, a total of 6022 proteins were statistically validated in at least 12 out of the 16 samples (FDR 0.01). The statistical significance of differences in protein abundance among the strains was determined by Student’s *t* test at FDR of 3%. In total, the concentrations of 1320 proteins were significantly different between the NO-resistant strain and the NO-susceptible strain; the concentration of 474 proteins was different between these strains treated with NaNO_2_ (2853+NO and 2856+NO), 850 between 2853 and 2853+NO, and 122 between 2856 and 2856+NO (Table S3 and Figure S2). These results reveal natural intrinsic differences in the proteome between the resistant and susceptible strains and show that the NO-resistant strain more actively modulates its proteome in response to the NO challenge than the NO-susceptible one.

### 3.4. The Nitrosative Challenge Negatively Modulated Antioxidant Proteins of NO-Susceptible L. braziliensis Strain

**Figure 4:**
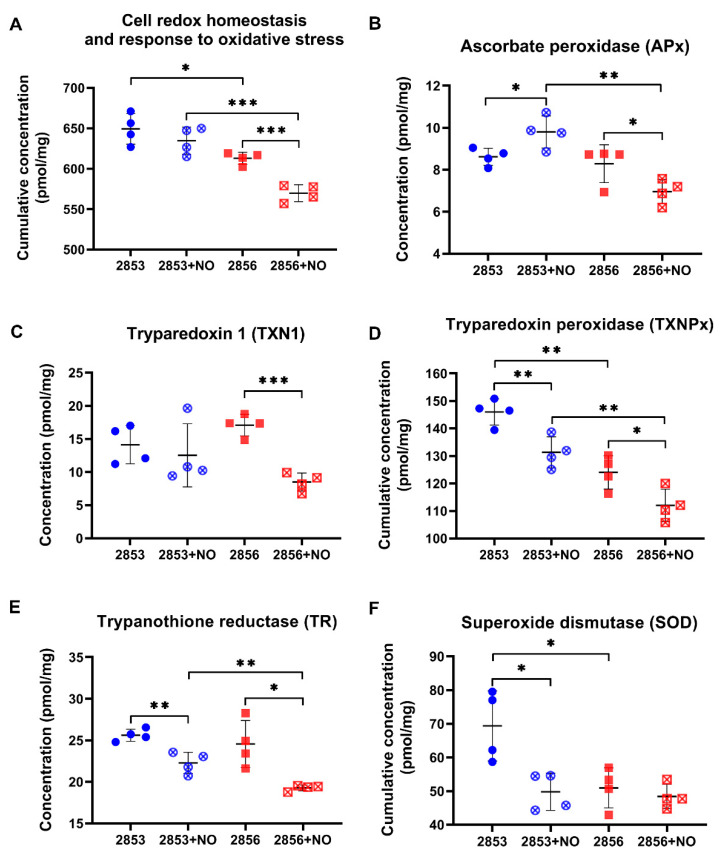
The antioxidant proteins of NO-susceptible *L. braziliensis* strain are negatively modulated after the nitrosative challenge. (**A**) Cumulative concentration of all identified proteins involved in biological processes of “*cell redox homeostasis and response to oxidative stress*”. Each symbol shows the total sum of the concentration values of proteins involved in those processes in each of the four biological replicates. Concentrations of specific proteins involved in response to oxidative stress: (**B**) ascorbate peroxidase (APx); (**C**) tryparedoxin 1 (TXN1); (**D**) tryparedoxin peroxidase (TXNPx); (**E**) trypanothione reductase (TR); and (**F**) superoxide dismutase (SOD). Graphs represent mean ± SD of four independent experiments. Statistical differences by Student’s *t* test (* *p* < 0.05; ** *p* < 0.01; *** *p* < 0.001). 2853: NO-resistant strain; 2853+NO: NO-resistant strain challenged with ^1^/_5_ IC_50_/4 h NaNO_2_; 2856: NO-susceptible strain; and 2856+NO: NO-susceptible strain challenged with ^1^/_5_ IC_50_/4 h NaNO_2_.

As the 2853 and 2856 strains have different responses to pro-oxidant and antioxidant stimuli and exhibit significant differences in their proteomes, we analyzed the abundance levels of proteins involved in the response to oxidative stress and in the maintenance of parasites’ redox homeostasis in our proteomics dataset. First, we observed that cumulative concentration of proteins involved in those processes is significantly higher in NO-resistant parasites than in the NO-susceptible. Interestingly, upon nitrosative challenge, the resistant 2853 strain maintains higher concentrations of those proteins, while the susceptible 2856 exhibits a significant decrease of them (Figure 4A). 

We also analyzed the concentration of specific proteins that are directly involved in the response to oxidative stress. The abundance of ascorbate peroxidase (APx), a heme-containing enzyme that catalyzes the conversion of H_2_O_2_ into water, was oppositely modulated by NO challenge, increasing in the 2853 strain and decreasing in the 2856 (Figure 4B). The NO-susceptible strain challenged with NaNO_2_ also exhibited a significant decrease in the absolute concentration of enzymes related to trypanothione-dependent hydroperoxide metabolism, such as tryparedoxin 1 (TXN1) (Figure 4C), tryparedoxin peroxidase (TXNPx) (Figure 4D), and trypanothione reductase (TR) (Figure 4E). Despite the impairment of TXNPx and TR abundance caused by nitrosative stress in NO-resistant parasites, the concentration of these proteins was higher than that observed in the NO-susceptible ones challenged with NO donor (Figure 4D,E). Interestingly, in conditions without NO challenge, NO-resistant strain also exhibited significant higher concentration of superoxide dismutase (SOD) when compared to the NO-susceptible one; however, treatment with NaNO_2_ significantly decreased cumulative SOD concentration to levels detected in 2856 strain (Figure 4F). These results show that upon NO challenge, the NO-resistant strain is more able to sustain the levels of trypanothione system’s enzymes than the NO-susceptible strain.

### 3.5. NO-Resistant L. braziliensis Strain Challenged with NO Increases the Abundance of Enzymes Involved in the Glutathione Pathway

**Figure 5:**
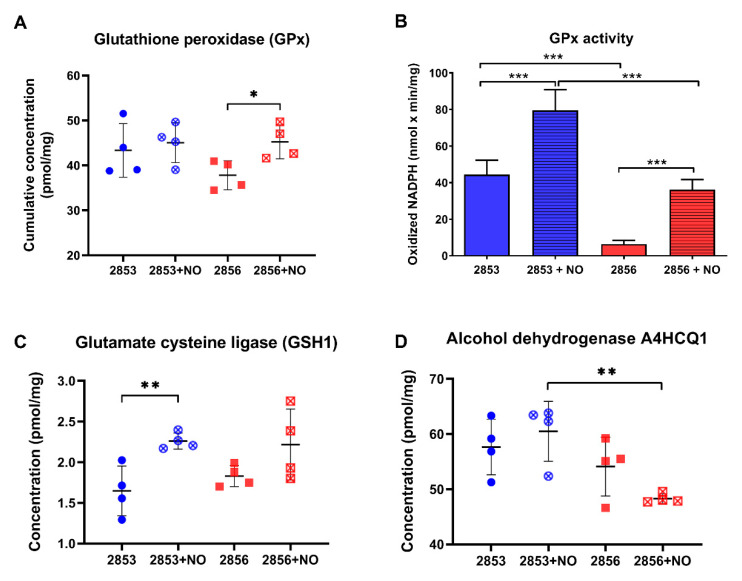
The NO-resistant strain increases glutathione metabolism enzymes in response to ^•^NO challenge. (**A**) Cumulative concentration of all glutathione peroxidases (GPx) identified in our dataset. Each symbol shows the total sum of the GPx concentration values in each of the four biological replicates. (**B**) GPx activity was measured in parasite homogenates. (**C**) Absolute concentration of glutamate cysteine ligase (GSH1) in each strain. (**D**) Absolute concentration of alcohol dehydrogenase A4HCQ1 in each strain. Graphs represent mean ± SD of four independent experiments. Statistical differences by Student’s *t* test (* *p* < 0.05; ** *p* < 0.01; *** *p* < 0.001). 2853: NO-resistant strain; 2853+NO: NO-resistant strain challenged with ^1^/_5_ IC_50_/4 h NaNO_2_; 2856: NO-susceptible strain; and 2856+NO: NO-susceptible strain challenged with ^1^/_5_ IC_50_/4 h NaNO_2_.

Since glutathione (GSH) is involved in the antioxidant response against oxidative and nitrosative stresses in eukaryotes [34,35], we analyzed the abundance levels of proteins involved in its metabolism in our dataset. Interestingly, although differences in glutathione peroxidase (GPx) cumulative abundance levels were detected only in NO-susceptible parasites challenged with NO donor (Figure 5A), the activity of this enzyme was ~7-fold higher in NO-resistant parasites than in NO-susceptible ones. Additionally, after exposure to theNO donor, GPx activity was significantly upregulated in both strains but was significantly higher in NO-resistant strain (Figure 5B). In addition, upon NO challenge, the concentration of glutamate-cysteine ligase (GSH1), which is an important enzyme responsible for the de novo synthesis of GSH, was significantly increased in NO-resistant parasites (Figure 5C). GSH may also interact directly with NO producing S-nitrosoglutathione (GSNO) [36,37,38], which can be decomposed by GSNO reductases to oxidized glutathione (GSSG). Alcohol dehydrogenase class III enzymes have GSNO reductase activity and function as a protection against nitrosative stress [39,40]. Notably, we found an alcohol dehydrogenase class III significantly decreased in the 2856 NO-susceptible strain challenged with NaNO_2_, in relation to 2853 treated with NO donor (Figure 5D). Altogether, these results suggest that GSH metabolism may be triggered by NO in *L. braziliensis* strains, especially in NO-resistant ones. In addition, alcohol dehydrogenase class III could also have GSNO reductase activity in *L. braziliensis,* and its downregulation may contribute to the inefficient NO detoxification in NO-susceptible parasites.

### 3.6. Uptake of Glucose Analog and G6PDH Protein Levels Increase in NO-Resistant L. braziliensis Strain after the Nitrosative Challenge

**Figure 6:**
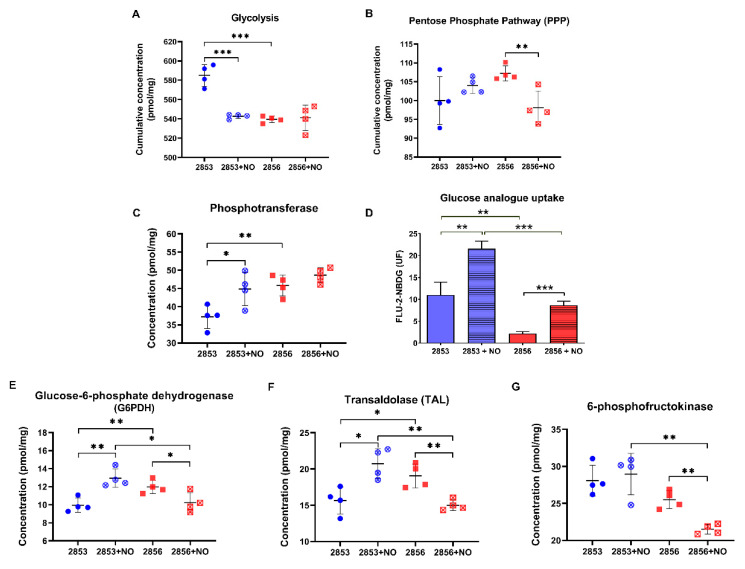
2-NBDG uptake and G6PDH activity increase in NO-resistant *L. braziliensis* strain after the nitrosative challenge. Cumulative concentration of all the identified proteins involved in (**A**) glycolysis and (**B**) pentose phosphate pathway (PPP). Each dot represents the total sum of the concentration values of proteins involved in those processes in each of the four biological replicates. Absolute protein concentration of (**C**) phosphotransferase; (**E**) glucose-6-phosphate dehydrogenase (G6PDH); (**F**) transaldolase; and (**G**) 6-phosphofructokinase-1. (**D**) 2-NBDG uptake after parasite incubation for 30 min at 25 °C. Median values were obtained by subtraction of fluorescence at 4 °C. Graphs represent mean ± SD of at least four independent experiments. Statistical differences by Student’s *t* test (* *p* < 0.05; ** *p* < 0.01; *** *p* < 0.001). 2853: NO-resistant strain; 2853+NO: NO-resistant strain challenged with ^1^/_5_ IC_50_/4 h NaNO_2_; 2856: NO-susceptible strain; and 2856+NO: NO-susceptible strain challenged with ^1^/_5_ IC_50_/4 h NaNO_2_.

Because we observed that NO-resistant strain exhibits more robust antioxidant defenses and responds more efficiently to the nitrosative challenge, mainly through the GSH metabolism, we analyzed if proteins involved in GSH reduction are also modified in those parasites, particularly those committed to NADPH production. First, we examined the abundance of enzymes involved in glycolysis and PPP (Figures S3 and S4). Cumulative concentration of glycolytic enzymes was significantly higher in the 2853 strain than in 2856. Resistant strain significantly diminished protein concentration levels of glycolytic enzymes upon NO challenge, but the glycolytic pathway titers remained similar in NO-susceptible parasites after NO exposure (Figure 6A). Notably, cumulative concentration of PPP enzymes was significantly reduced in NO-susceptible parasites but not in NO-resistant ones in response to NO stimulus (Figure 6B). Analysis of phosphotransferase, the first enzyme of the glycolytic pathway, revealed that 2856 strain has higher protein abundance in comparison to 2853; however, after nitrosative challenge, NO-resistant parasites exhibited a significant increase in this enzyme, while the same did not happen in NO-susceptible ones (Figure 6C). Interestingly, such increase is reflected in significantly elevated glucose analogue uptake in those parasites, which was ~2.5-fold higher in 2853 strain than in 2856 after NO exposure (Figure 6D). 

Glucose consumed by parasites is rapidly converted to glucose 6-phosphate, which can follow the glycolytic pathway or be driven to PPP and used for maintaining the NADPH pool. In fact, we observed a significant increase in the concentration of G6PDH in the NO-resistant strain upon NO challenge, whereas the enzyme concentration was reduced in the NO-susceptible one under the same stress condition (Figure 6E). In addition, a significant increase in transaldolase (TAL), an enzyme of non-oxidative branch of PPP, was observed in NO-resistant parasites, while a significant reduction of this protein was detected in the NO-susceptible ones (Figure 6F). Analysis of protein abundance of other glycolytic enzymes supports the idea that glucose 6-phosphate may be entering the PPP pathway in NO-resistant parasites (Figures S3 and S4). Particularly, although the levels of 6-phosphofructokinase-1, an important enzyme to glycolytic pathway, were higher in the NO-resistant parasites than in NO-susceptible ones, they were not modulated by the NaNO_2_ treatment (Figure 6G). 

### 3.7. Nitrosative Challenge Increases D-Lactate Dehydrogenase Abundance in NO-Resistant L. braziliensis Strain 

**Figure 7:**
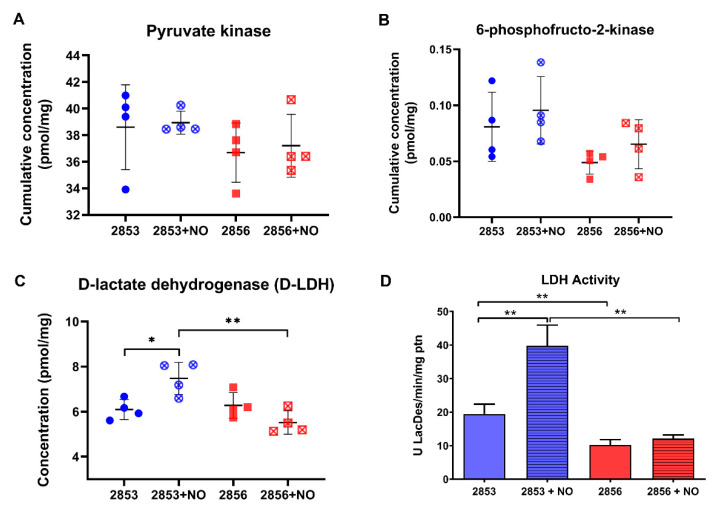
Nitrosative challenge increases D-lactate dehydrogenase (D-LDH) abundance in NO-resistant *L. braziliensis* strain. (**A**) Protein concentration of pyruvate kinase; (**B**) cumulative concentration of 6-phosphofructo-2-kinase; (**C**) protein concentration of D-lactate dehydrogenase (D-LDH); and (**D**) LDH activity in parasite homogenates. Graphs represent mean ± SD of at least four independent experiments. Statistical differences by Student’s *t* test (* *p* < 0.05; ** *p* < 0.01). 2853: NO-resistant strain; 2853+NO: NO-resistant strain challenged with ^1^/_5_ IC_50_/4 h NaNO_2_; 2856: NO-susceptible strain; and 2856+NO: NO-susceptible strain challenged with ^1^/_5_ IC_50_/4 h NaNO_2_.

As NO challenge seems to positively regulate the first steps of glycolysis, we were interested in analyzing the status of the enzymes at the pathway exit. The concentration levels of pyruvate kinase, the only known regulated glycolytic enzyme in *Leishmania*, did not suffer significant alterations upon exposure to NO (Figure 7A). In addition, the allosteric regulator of pyruvate kinase, 6-phosphofructo-2-kinase, the enzyme responsible for fructose-2,6-bisphosphate biosynthesis, did not suffer significant changes upon exposure to NO (Figure 7B). Intriguingly, we observed a significant increase in the concentration and activity of D-lactate dehydrogenase (D-LDH) in NO-resistant strain upon NO exposure (Figure 7C,D). Indeed, LDH activity was increased 2-fold in NO-resistant parasites after nitrosative challenge and it was 3.3-fold higher in NO-resistant-challenged parasites than in NO-susceptible-challenged ones (Figure 7D). 

### 3.8. Nitrosative Challenge Impairs Mitochondrial O_2_ Consumption by L. braziliensis Strains

**Figure 8:**
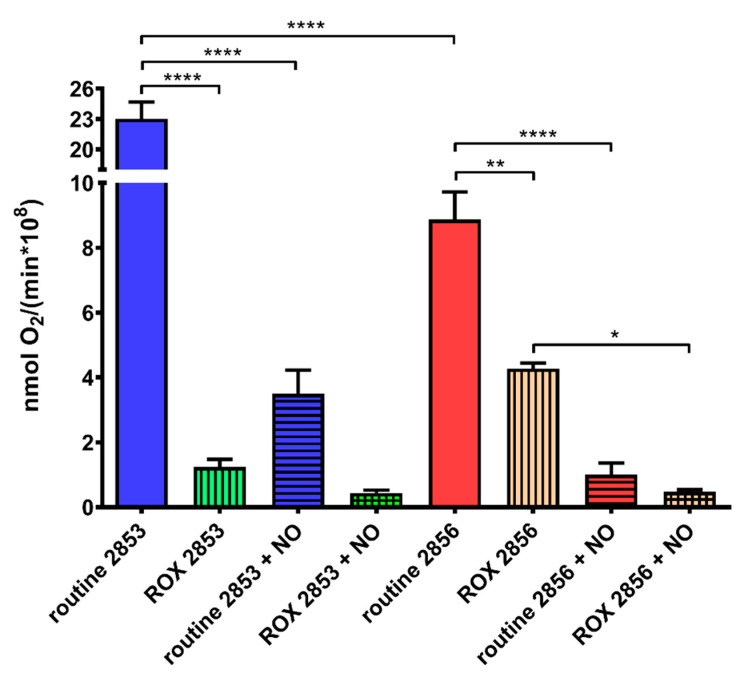
Nitrosative challenge impairs mitochondrial O_2_ consumption by *L. braziliensis* strains. Parasites were incubated in the respiration buffer at 25 °C to evaluate the O_2_ uptake under routine condition and after the addition of 2 μM AA (ROX state). Graphs represent mean ± SD of four independent experiments. Significance of differences between treatments were determined by two-way ANOVA followed by Tukey’s multiple comparisons test (* *p* < 0.05; ** *p* < 0.001; and **** *p* < 0.0001). 2853: NO-resistant strain; 2853+NO: NO-resistant strain challenged with ^1^/_5_ IC_50_/4 h NaNO_2_; 2856: NO-susceptible strain; and 2856+NO: NO-susceptible strain challenged with ^1^/_5_ IC_50_/4 h NaNO_2_.

Mitochondrial respiration and, specifically, proteins involved in oxidative phosphorylation (OXPHOS) are inhibited by NO due to competition with oxygen [41]. Here, we observed that oxygen consumption in routine condition is 61.5% lower in 2856 strain than in 2853. Additionally, the mitochondrial respiration is more affected by NaNO_2_ treatment in NO-susceptible parasites than in NO-resistant. Thus, although nitrosative stress induced a significant decrease in both strains, NO-resistant parasites can maintain oxygen consumption 1.7-fold higher than NO-susceptible (Figure 8). Interestingly, ROX state, which indicates mitochondrial-independent oxygen consumption, increased~3.5-fold in 2856 strain when compared to 2853, suggesting higher ROS production by NO-susceptible parasites. Besides that, the NO exposure decreased the oxygen consumption to similar levels during ROX state in both strains (Figure 8). 

**Figure 9:**
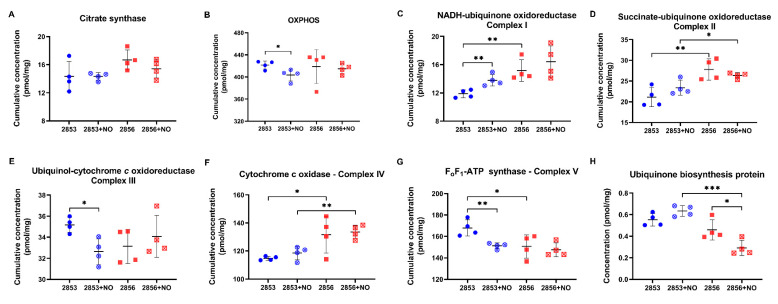
Proteins of mitochondrial oxidative phosphorylation (OXPHOS) are differentially modulated in *L. braziliensis* strains. (**A**) Cumulative concentration of all the identified proteins as citrate synthase. Cumulative concentration of all the identified proteins involved in (**B**) OXPHOS; (**C**) NADH-ubiquinone oxidoreductase (complex I); (**D**) succinate ubiquinone oxidoreductase (complex II); (**E**) ubiquinol:cytochrome c oxidoreductase (complex III); (**F**) cytochrome c oxidase (complex IV); and (**G**) F_o_F_1_-ATP synthase (complex V). Each dot represents the total sum of the concentration values of proteins involved in those processes or complexes in each of the four biological replicates. (**H**) Absolute protein concentration of ubiquinone biosynthesis protein. Graphs represent mean ± SD of at least four independent experiments. Statistical differences by Student’s *t* test (* *p* < 0.05; ** *p* < 0.01; and *** *p* < 0.001). 2853: NO-resistant strain; 2853+NO: NO-resistant strain challenged with ^1^/_5_ IC_50_/4 h NaNO_2_; 2856: NO-susceptible strain; and 2856+NO: NO-susceptible strain challenged with ^1^/_5_ IC_50_/4 h NaNO_2_.

Based on these results, the abundance of mitochondrial complexes, as well as the concentration levels of several proteins, were analyzed. First, we observed that there were no differences in the concentration of citrate synthase, an enzyme associated with mitochondrial integrity, among the experimental groups (Figure 9A). Notably, we observed a significant decrease in the accumulated abundance of the proteins involved in OXPHOS in the NO-resistant parasites after the NO challenge (Figure 9B). Intriguingly, the protein concentration of complexes I, II, and IV is significantly higher in NO-susceptible parasites than in NO-resistant, and the exposure to nitrosative stress does not affect the abundance of these proteins (Figure 9C,D,F). In contrast, protein concentration of complex V is significantly lower in NO-susceptible parasites than in NO-resistant ones (Figure 9G). Interestingly, upon nitrosative challenge, parasites of 2853 strain increase the concentration of molecules related to complex I and decrease the abundance of complexes III and V (Figure 9C,E,G). Ubiquinone acts as an electron carrier from complexes I and II to complex III. Interestingly, we found that the concentration of a protein that participates in the biosynthesis of this molecule is significantly decreased in NO-susceptible parasites during nitrosative condition (Figure 9H), suggesting that 2856 strain may be unable to maintain the ubiquinone pool under NO exposure. 

## 4. Discussion

To complete its life cycle, *Leishmania* must overcome several barriers found in the vertebrate host, such as increased temperature (25 °C in insect vector to 37 °C in mammalian host), pH acidification in phagolysosome, and changes of available carbon sources [42]. Moreover, the parasite must survive the oxidative burst and NO production, two main microbicidal mediators that started after macrophage activation [43]. Although the host immune response is directly related to the prognosis of leishmaniasis [44], intrinsic virulence features of *Leishmania* strains are also decisive for infection and clinical outcome [21]. In the present study, we evaluated two strains of *L. braziliensis* with polarized phenotype of susceptibility or resistance to NO, which were also associated with responsiveness or refractoriness to antimony treatment [17,20]. Aspects related to nitrosative and oxidative stresses suffered by the parasites during host cells infection, in addition to a deep analysis of parasites’ proteome-mediated mechanisms for resisting to NO were considered. It is important to point out that during the very initial steps of infection, promastigotes are subjected to the early oxidative burst triggered into the innate immunity cells -at the same time host and effectors- in response to the invasion. To cope with this, promastigotes should display their repertoire of antioxidant responses. Depending on the success during such very early responses, parasites will survive, and differentiate, determining the successful parasite colonization and further persistence. Our proteomics and biochemical data on promastigotes challenged with NaNO_2_ would reflect these very initial parasite responses.

First, in vitro infection allowed for the identification of differences between both strains, corroborating the higher infection capability of NO-resistant parasites, both in terms of number of infected macrophages, as well as in number of amastigotes per cell. Our findings were similar to those of Souza et al. [20], who used human macrophages for in vitro evaluation of *L. braziliensis* infection. Interestingly, we also observed that both strains trigger host microbicidal responses through increased production of ROS and RNS, with levels exacerbated in the infection produced by NO-resistant strain. In cutaneous leishmaniasis, the development of a non-healing course of infection has been linked to an immunosuppressive profile, with increased arginase 1 activity [45], a cytosolic enzyme that favors parasites’ antioxidant metabolism and that, at the same time, competes with iNOS for the common substrate L-arginine, decreasing NO production [46,47]. Although Costa et al. [21] demonstrated that this process also occurs during in vivo infection of BALB/c mice with the NO-resistant *L. braziliensis* strain, it is only elicited in late infection times (seven weeks post-infection). All together, these data suggest that NO-resistant parasites are endowed with specific mechanisms to evade host defenses, raising a question about the phenotypic adaptations that allow this strain to survive in the presence of high concentrations of toxic molecules, especially during early infection course. 

According to previous studies, *L. braziliensis* amastigotes survive and replicate much better in the absence of ROS, identifying these molecules as important regulators of the parasite proliferation inside the host cells [48]. In addition, supplementation of cell cultures with pro-oxidants and antioxidants modulates *Leishmania* infection, resulting in a reduced parasite load in stressful conditions [49]. Moreover, sensitivity to distinct reactive species may vary in the parasite stages, with H_2_O_2_ being more toxic to *Leishmania* amastigotes than O_2_^•−^ [43,50]. In line with those observations, infection with the NO-susceptible strain reproduces all phenotypes previously described, including the high sensitivity to H_2_O_2_ (comparing the infection index of catalase- and SOD-treated cells). In contrast, the infection with NO-resistant parasites has unique features; only increased nitrosative stress derived from NaNO_2_-treated macrophages was able to downregulate the infection by this strain. Resistant parasites also appear to be unaffected by H_2_O_2_ since neither this reactive species (even with the increase in RNS production) nor the presence of catalase were able to change the infection index. In contrast, O_2_^•−^ metabolism might be essential for NO-resistant strain because only SOD-treated cells showed reduction in the parasite load. The use of tempol, an antioxidant able to promote O_2_^•−^ dismutation at rates similar to SOD, pointed to the dual role of this free radical to control the infection, highlighting differences in the pathogenesis of cutaneous leishmaniasis caused by different species of *Leishmania* [49,51,52].

To shed light on the molecular mechanisms underlying the NO-resistant phenotype in *L. braziliensis*, we performed an unbiased and comprehensive quantitative analysis of parasites’ proteome. Notably, differences in total protein per cell and absolute protein concentrations clearly distinguished NO-resistant from NO-susceptible parasites, corroborating the notion that there are natural intrinsic differences at proteome level among *L. braziliensis* strains circulating in a same geographical region [9]. In addition, we demonstrated that the rapid modulation of NO-resistant parasites’ proteome upon NO challenge involves an increase in total protein content, which is suggestive of ploidy alterations in response to nitrosative stress. In line with this proposal, recently it was reported that aneuploidy in *L. donovani* is followed by proteome modulation and could explain metabolic differences between strains [53]. Aneuploidy and karyotypic mosaicism are common in *Leishmania* spp., and such genome plasticity allow parasites to explore fitness possibilities for survival [54,55,56]. In *Leishmania*, aneuploidy is a species- and strain-specific trait that varies according to external stimuli, including drug pressure, enabling rapid adaptation to hostile conditions [57,58,59,60]. However, aneuploidy may also result in metabolic alterations that lead to oxidative and proteotoxic stresses [61]. Interestingly, we observed that in contrast to NO-resistant strain, the NO-susceptible parasites naturally (without NO stimulus) present a protein content higher than expected and are not able to modulate it upon NO challenge, suggesting that NO-susceptible parasites are “naturally stressed” and that proteome (and genome) plasticity in these parasites probably reached a limit. Remarkably, the deep proteomics approach conducted in this study allows one to clearly differentiate resistant parasites from susceptible ones and reveals the subset of proteins that explain those phenotypes.

Proteomic profiling of *L. donovani* parasites adapted to sub-lethal doses of NO donor showed the upregulation of several parasite’s proteins involved in the ROS detoxification pathway, suggesting a cross-resistance to both nitrosative and oxidative stresses [62]. Accordingly, we observed a positive modulation in the concentration of proteins associated with *“cell redox homeostasis and response to oxidative stress*” in NO-resistant parasites, even in the condition without the NO donor. In addition, the challenge with NaNO_2_ specifically affected the concentration of APx, increasing protein abundance in NO-resistant parasites and decreasing in NO-susceptible ones. Sardar et al. [61] also demonstrated that exposure of *L. donovani* promastigotes to ROS or RNS elevates APx protein abundance up to 2.5-fold, whereas combination of both stresses produced an additive effect, increasing this protein in 3.2-fold. Additionally, ROS-inducible APx of *L. amazonensis* is essential for parasite infectivity, replication, and virulence in vitro and in vivo models [63]. Collectively, these data support the idea that NO-resistance in *L. braziliensis* is associated with APx increased abundance as a response to stressful conditions, which also explains the successful intracellular replication of NO-resistant parasites in macrophages overproducing H_2_O_2_. Interestingly, *L. braziliensis* APx-overexpressing parasites have an 8-fold increase in the antimony-resistance index [64]. As *L. braziliensis* NO-resistant parasites used here are also refractory to antimony treatment, our data reinforce the idea that APx could participate in both NO- and Sb-resistance.

Regarding the T(SH)_2_/TR system, we observed a decrease in TR abundance after NO challenge. Although this phenomenon occurred to a lesser extent in NO-resistant parasites, such reduction indicates that other mechanisms are concurring to maintain the pool of reducing intermediates. Despite the fact that T(SH)_2_ is a much more efficient scavenger of hydroperoxides than other thiols present in trypanosomatids [65], all these protozoa possess glutathione-dependent enzymes [66]. Susceptibility of different *Leishmania* species to the NO donor S-nitroso-N-acetyl-D,L-penicillamine (SNAP) was inversely correlated with the levels of GSH but not with their total thiol content (including T(SH)_2_ levels) [67]. In addition, *L. donovani* showed ~2.9-fold upregulation of glutathione peroxidase-like protein in detrimental to TR in response to nitrosative stress [62]. In agreement with those observations, our results show that *L. braziliensis* NO-resistant parasites have higher concentrations of proteins involved in GSH biosynthesis pathway, particularly GSH1, and GPx activity than NO-susceptible parasites upon NO exposure. Such increased levels can avoid the toxic effects of nitrosative stress and oxidative burst found after treatment with NO donor, or during host infection. Additionally, we cannot rule out the role of GSH pool in regeneration of ascorbate, a cofactor of APx, since in several organisms this is the main ascorbate recycling system [68]. Although in trypanosomatids this process seems to be achieved via T(SH)_2_ [69], further analyses should be performed to evaluate the activity of GSH in *L. braziliensis* parasites, especially in NO-resistant ones.

In *L. donovani,* the response to oxidative stress requires a rapid metabolic reconfiguration of glucose metabolism, involving a shift from glycolysis toward PPP for replenishment of NADPH pool when parasites are exposed to oxidants. mRNA and protein levels of PPP enzymes of the oxidative and nonoxidative branch, such as G6PDH and transaldolase, were up-regulated in promastigotes exposed to sublethal doses of pro-oxidants, while the viability of promastigotes treated with G6PD inhibitor and sublethal doses of ROS was restored by coincubation with N-acetyl cysteine or GSH [70]. In addition, cell lines overexpressing G6PDH and transaldolase are also more resistant to antimonial, amphotericin B, and miltefosine [70]. In agreement, our dataset indicates that NO resistance in *L. braziliensis* also involves a rapid shift in glucose metabolism from glycolysis to PPP in response to nitrosative stress, without the detriment of glycolysis per se. Protein concentration of G6PDH and transaldolase were increased in NO-resistant parasites and decreased in NO-susceptible ones in response to the NO challenge. Such increase in protein concentration enables NO-resistant parasites to replenish intracellular NADPH levels and, consequently, the GSH pool, to maintain its cellular redox balance. The proteome of promastigotes of *L. infantum* strains resistant to NO also showed increased abundance of G6PDH, while amastigotes of *L. infantum* cell lines selected for NO-resistant exhibited overexpression of 6-phosphogluconate dehydrogenase mRNA levels [23,71], reinforcing the notion that the PPP is an indispensable pathway for resistance to nitrosative stress. 

Intriguingly, we observed a significant increase in the concentration of D-LDH in NO-resistant parasites after exposure to NO, and such increase was accompanied by increased LDH enzymatic activity. In *L. major,* D-lactate produced via methylglyoxal metabolism would be converted to pyruvate by a D-LDH, with the resulting molecule feeding the tricarboxylic acid cycle (TCA) and other pathways [72,73,74]. As the methylglyoxal is a toxic glycolytic metabolite, we hypothesize that the increase in glucose uptake and, consequently, in the production of methylglyoxal may occur in response to nitrosative stress in NO-resistant parasites challenged with NO, leading to upregulation of D-LDH abundance and activity in these parasites. However, further assays need to be done to demonstrate the accumulation of that metabolite in parasites under nitrosative stress. Interestingly, and supporting once again the idea that NO- and drug-resistance are related, recently it was observed that transcript levels coding for D-LDH increased 56-fold in a *L. donovani* cell line selected for paromomycin resistance and that wild-type parasites transfected with *D-LDH* acquired a significant resistance against the drug [75]. 

It has been suggested that a metabolic shift from glycolysis to mitochondrial respiration is detrimental for *L. mexicana* virulence in vivo, leading to high ROS production and increased sensitivity of parasites to NO. Therefore, the maintenance of glucose uptake would be an advantage in the oxidative environment of the phagolysosome [76]. Accordingly, in NO-resistant parasites, apart the complex I, the concentration of all components of mitochondrial electron transport system was not modified or was even diminished (complexes III and V) after NO challenge. Such results may explain the drastic decrease in oxygen consumption by NO-resistant strain upon NO exposure. This scenario is extremely interesting and raises several possibilities about the metabolic adaptations suffered by *L. braziliensis*. For an efficient microbicidal response, macrophages need to activate both NADPH oxidases and iNOS, which can cause hypoxia conditions and lead to expression of hypoxia-inducible factor-1α (HIF-1α) by host cells [45]. In patients with ATL caused by *L. braziliensis,* the expression of this transcription factor has already been described, suggesting hypoxia participation during cutaneous and mucocutaneous clinical outcomes [77]. As described by Degrossoli et al. [78], low oxygen tension, derived from enhanced ROS generation, also leads to the reduction of intracellular parasites in *L. amazonensis*-infected macrophages. Together, these data may suggest that the phagolysosomal environment, rich in NO and ROS, is not an attractive place to perform OXPHOS and derived metabolisms since they are dependent on oxygen availability. A model reconstruction of energy metabolism in *L. infantum* suggested a reduction in oxygen intake in the amastigote scenario, in comparison to promastigotes, probably indicating the adaptation of amastigote metabolism to hypoxic environment of the macrophage [79]. Hence, the observed increase in glucose uptake and the decrease in mitochondrial oxygen consumption after NO challenge could reflect the adaptations that amastigote forms of NO-resistant *L. braziliensis* should undergo seek to survive in host cells. In addition, complex I would be increased to maintenance of NADH/NAD^+^ ratio since complex I seems to conserve all subunits containing the redox centers required to ubiquinone reduction [80,81].

## 5. Conclusions

Together, our data show that NO resistance in *L. braziliensis* involves rapid remodeling of the parasites’ proteome, resulting in increased protein content as well as in increased GSH metabolism, higher levels of glucose consumption, elevated abundance of PPP enzymes, and lower mitochondrial respiration, all of which can contribute to thiol and NADPH pool maintenance in these parasites, enabling them to successfully colonize and persist in host cells. 

## Data Availability

The mass spectrometry proteomics data have been deposited to the ProteomeXchange Consortium via the PRIDE [31] partner repository with the dataset identifier PXD029462.

## Supplementary Materials

The following supporting information can be downloaded at: https://www.mdpi.com/article/10.3390/antiox11020277/s1, Figure S1: Growth curves of *L. braziliensis* strains; Figure S2: Volcano plot representation of differences in protein concentration between NO-resistant and NO-susceptible *L. braziliensis* strains; Figure S3: Overview of the glycolysis pathway in *L. braziliensis* and identified enzymes; Figure S4: Overview of the pentose phosphate pathway (PPP) in *L. braziliensis* and identified enzymes; Table S1: Protein Groups identified; Table S2: Summary of general information about the proteomes of *Leishmania braziliensis* strains resistant or susceptible to NO; Table S3: Statistical differences among groups.
