# Supplementary material for: Nitric Oxide Resistance in Leishmania (Viannia) braziliensis Involves Regulation of Glucose Consumption, Glutathione Metabolism and Abundance of Pentose Phosphate Pathway Enzymes
Source: Antioxidants (Basel). 2022 Jan 29;11(2):277. doi: 10.3390/antiox11020277 (PMC8868067; doi:10.3390/antiox11020277)
Supplement: Supplementary file 1 [file antioxidants-11-00277-s001.zip › Supplementary Table S2.pdf]

Supplementary Table S2. Summary of general information about the proteomes of *Leishmania braziliensis* strains resistant or susceptible to NO.

	**2853** NO-resistant	**2853+NO** NO-resistant challenged with $NaNO_2$	**2856** NO-susceptible	**2856+NO** NO-susceptible challenged with $NaNO_2$
**Biological replicates**	4	4	4	4
**Peptides identified**	31019 ± 1288	37006 ± 846	41660 ± 759	42050 ± 1373
**Protein groups identified**	5707	5960	6002	6012
**Total protein per cell**	3.3 ± 0.11 pg	4.3 ± 0.08 pg	4.4 ± 0.2 pg	4.4 ± 0.1 pg
**Total protein molecules per parasite**	$5.42 \pm 0.16 \times 10^7$	$6.84 \pm 0.15 \times 10^7$	$7.10 \pm 0.28 \times 10^7$	$7.04 \pm 0.19 \times 10^7$
