# Supplementary figures and images for: Nitric Oxide Resistance in Leishmania (Viannia) braziliensis Involves Regulation of Glucose Consumption, Glutathione Metabolism and Abundance of Pentose Phosphate Pathway Enzymes
Source: Antioxidants (Basel). 2022 Jan 29;11(2):277. doi: 10.3390/antiox11020277 (PMC8868067; doi:10.3390/antiox11020277)

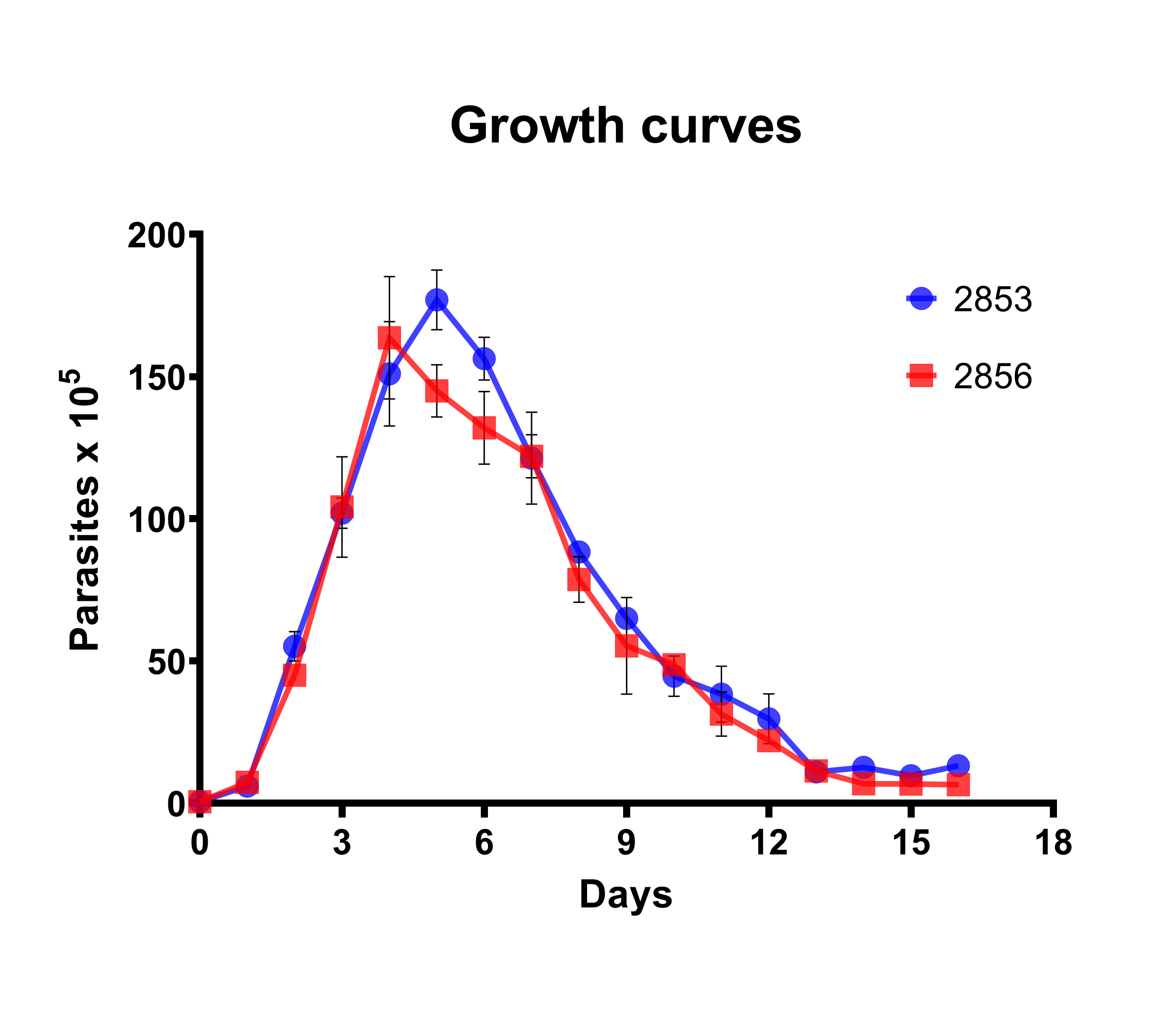

Supplement: Supplementary file 1 [file antioxidants-11-00277-s001.zip › Figure S1.tif]

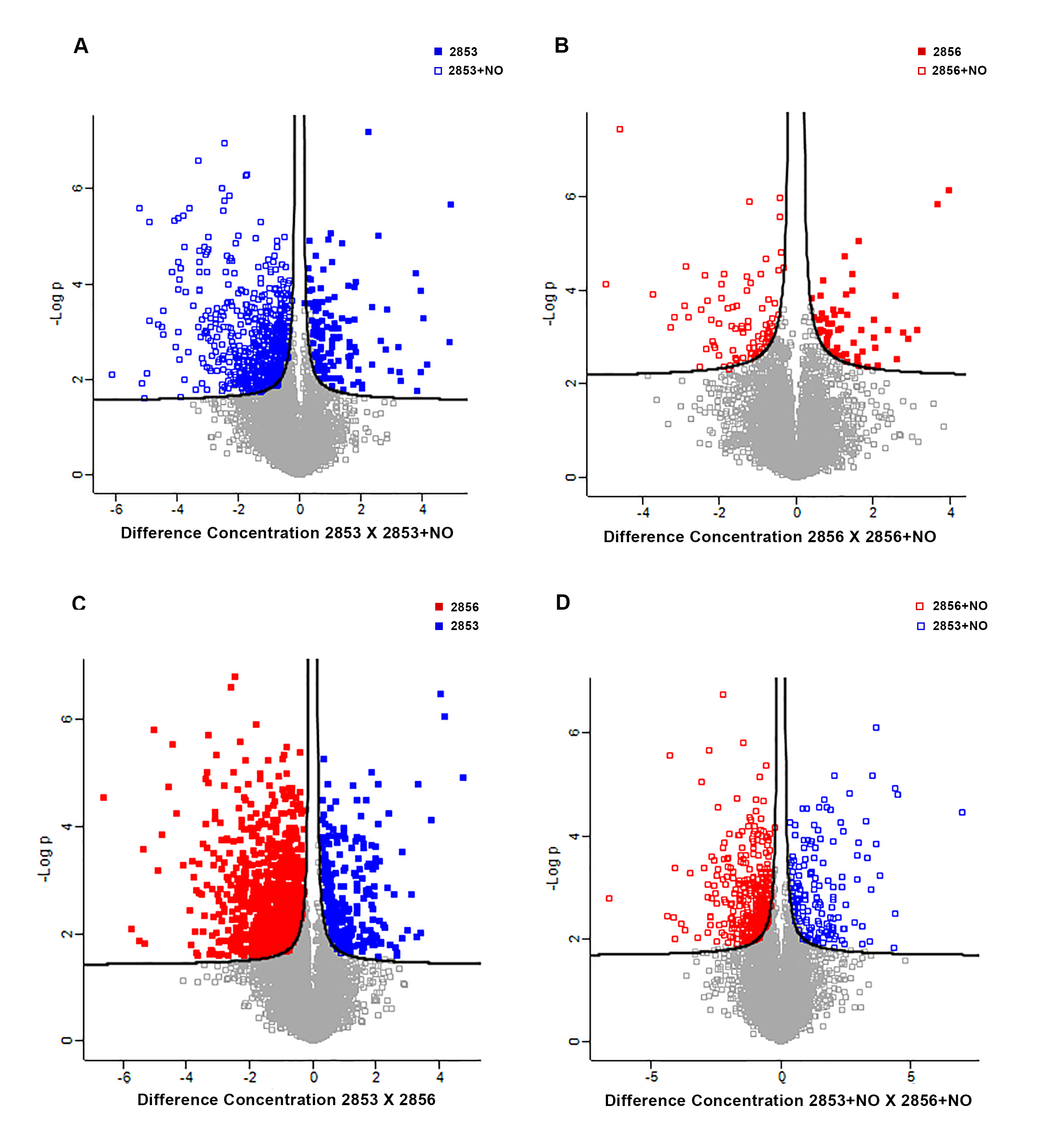

Supplement: Supplementary file 1 [file antioxidants-11-00277-s001.zip › Figure S2.tif]

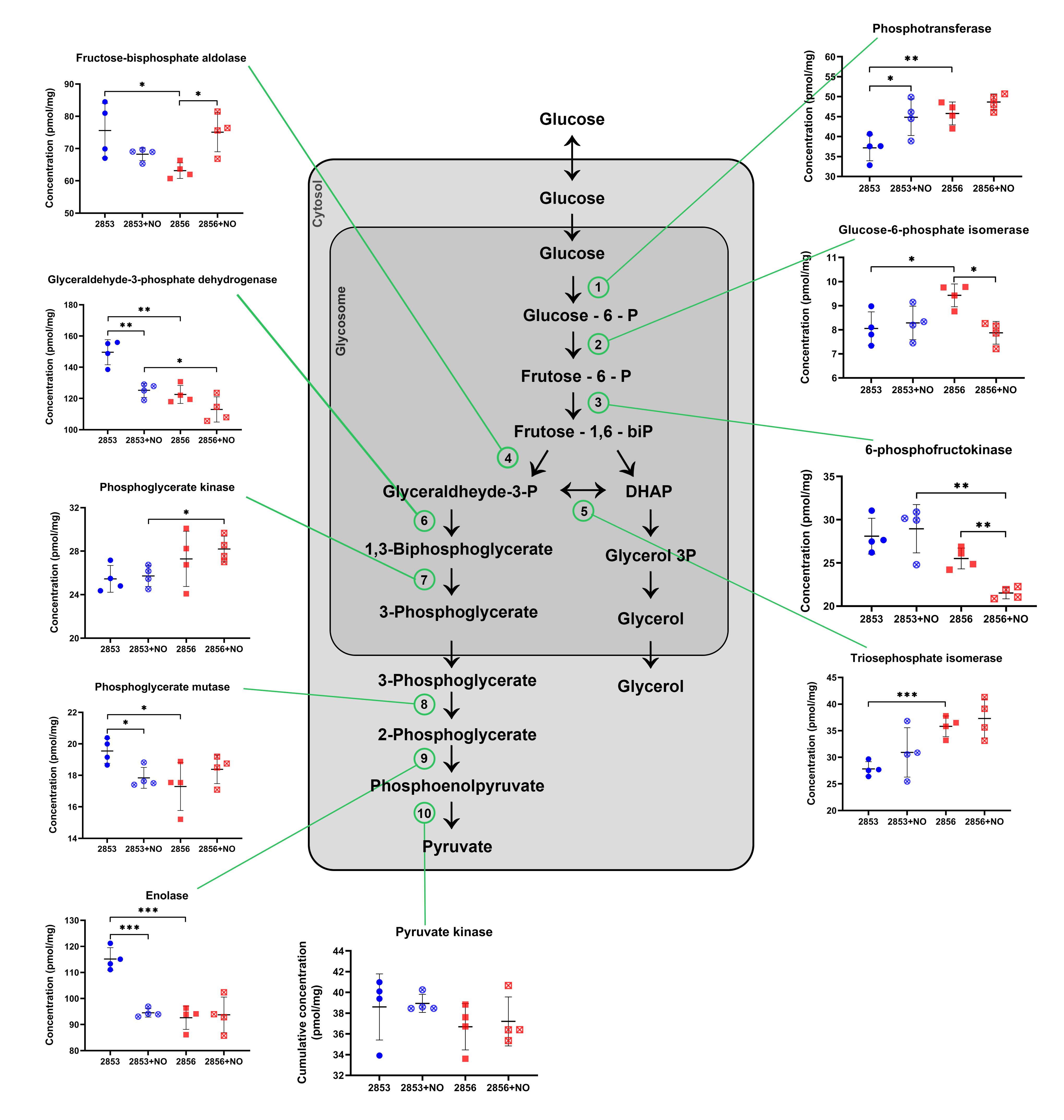

Supplement: Supplementary file 1 [file antioxidants-11-00277-s001.zip › Figure S3.tif]

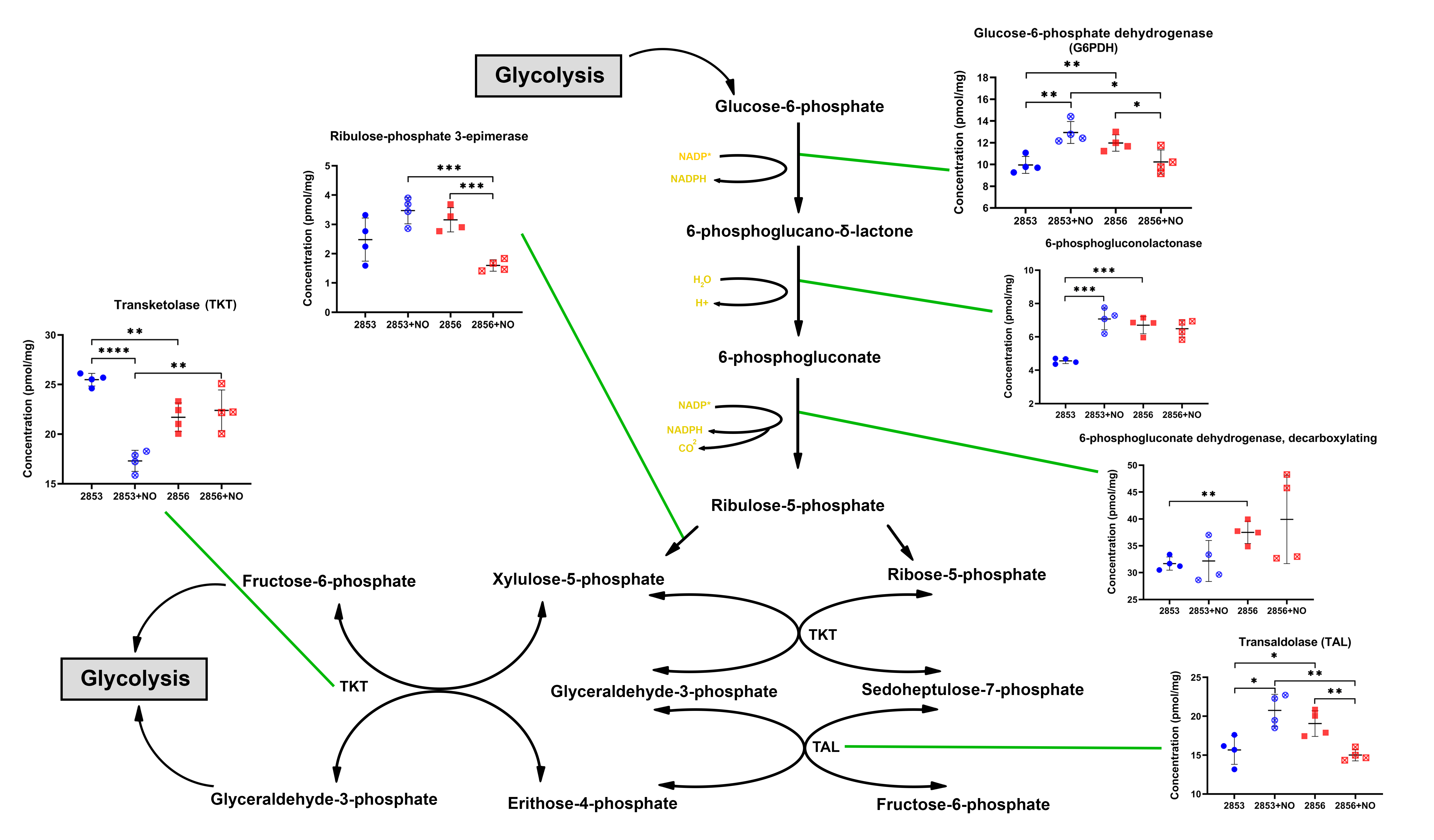

Supplement: Supplementary file 1 [file antioxidants-11-00277-s001.zip › Figure S4.tif]
